# Some Aspects of General Peritonitis—III

**Published:** 1908-04-25

**Authors:** 


### SOME ASPECTS OF GENERAL PERITONITIS.

### III.—THE COMPLICATION OF ILEUS.

One of the main difficulties, if not the very greatest, which is likely to be experienced in the treatment of cases of general peritonitis after operation, is that of getting the patient's bowels to act. This is due in many cases to the fact that no treatment is given until it is called for by the onset of symptoms. This is utterly wrong, because the onset of any symptom such as abdominal distension, which calls for the immediate administration of a purge or an enema is an indication that the intestinal wall has been affected by the general toxæmia and has begun to strike work; it is then too late to expect treatment, however energetic, to do any good. The sooner the bowels are opened after operation for general peritonitis, the sooner the patient is out of danger. Until that result has been achieved he must be regarded as having a very small chance of recovery. Statistics on cases of general peritonitis collected by different observers may vary on other points, but there is a singular unanimity on this one: that the sooner the bowels are induced to act, the less probability there is of the supervention of vomiting or distension, either of which is a grave complication of peritonitis.

Each case must, of course, be treated on its own merits, and it is difficult, nay, impossible, to lay down any definite or exact rule as to the nature of the aperient to be used, or the time after operation at which it should be administered. The following remarks must, therefore, be taken only as indicating a line of treatment which has been found satisfactory in a considerable number of cases.

The day after the operation an enema should be given; an ordinary soap-and-water enema of from 16 to 20 ounces is as good as any. If this is successful it is most satisfactory, because the evacuation is not accompanied by painful peristalsis which may distress the patient. If, however, a common enema produces no effect, substances which have a more irritating effect on the rectal mucous membrane may be successively tried, such as one ounce of turpentine or three drachms of confectio rutæ. The former may be made up with white of egg and sixteen ounces of water or gruel. With the latter the association of a similar quantity of chamomile infusion is an old-established and valuable formula. Fifteen grains of asafoetida is less well-known in this connection, but is recorded to have succeeded where both the others have failed.

But an enema alone, however good its result, is not sufficient: it must be combined with some form of purge given by the mouth. An enema only clears out the large intestine, and it is essential that the entire alimentary canal should be kept not only comparatively empty, but also in a state of moderate activity. For this purpose there is no drug to compare with calomel. It is true that castor oil is a very excellent purge in certain cases, but it is not a good one for a patient with a grave abdominal lesion, because he will almost certainly vomit it as soon as he has taken it. Calomel, on the other hand, gives rise to no nausea, and is easily retained, especially if it be administered in small doses at frequent intervals—one grain, for instance, every hour for six hours, or even more until the bowels act.

Saline aperients, admirable though they are in the after-treatment of an ordinary laparotomy, are not sufficiently urgent in their business to be successful in cases of peritonitis: and those who rest their hopes on the administration of saline purges, and them alone, after peritonitis are doomed to some great disappointments.

What is really badly needed is a satisfactory purgative which will act by absorption, and thus may be given subcutaneously. There is a great future for such a drug. Some have stated that saline aperients will act in this way; but experiment has not tended to uphold the truth of the statement. The advantage that such a drug would have is that it could be administered to a patient without fear of bringing on emesis. It is true that calomel rarely does this, but in the small percentage of cases where it does, one has nothing to fall back upon.

If this vigorous line of treatment is adopted from the beginning, it is possible to diminish appreciably the chance of abdominal distension owing to paralysis of the intestine, or ileus, as it is sometimes called. This is a condition in which the muscular coat of intestine shares in the general toxæmia, and peristalsis is completely inhibited. It may be taken as a positive indication of the onset of the last stage of peritonitis. If it has already occurred before the abdomen is opened, it is occasionally of value to inject into the intestine at the time of operation an ounce of a saturated solution of magnesium sulphate, and to sew up the puncture with a Lembert's suture. If, however, it comes on during the after-treatment of a case, the outlook must be regarded as being extremely grave. The introduction of a rectal tube to get rid of flatus may be of some assistance; and the administration of purges and enemata must again be vigorously pushed. But it most often happens that from this moment the patient goes rapidly down hill.

There is, however, one drastic measure which may save the patient's life, and that is to open the abdomen, bring out a coil of small intestine, and make a temporary artificial anus. The method is drastic because a patient with an artificial anus in the small gut is unable to live for more than six weeks or so; but in some cases the evacuation of retained contents from the small intestine enables it to recover its tone, and the paralysis disappears. The artificial anus can then be closed by another operation. Whenever, in these difficult cases of extensive peritonitis, by some means or another a copious evacuation is secured, it is particularly important to guard against sudden syncope, a complication which occasionally proves immediately fatal. A hypodermic injection of strychnine, with every care for the warmth of the patient, and the avoidance of straining as far as possible, are useful prophylactic measures.

